# Antidepressants and the risk of death in older patients with depression: A population-based cohort study

**DOI:** 10.1371/journal.pone.0215289

**Published:** 2019-04-15

**Authors:** Bianca Kollhorst, Kathrin Jobski, Jutta Krappweis, Tania Schink, Edeltraut Garbe, Niklas Schmedt

**Affiliations:** 1 Department of Biometry and Data Management, Leibniz Institute for Prevention Research and Epidemiology–BIPS, Bremen, Bremen, Germany; 2 Division of Outpatient Care and Pharmacoepidemiology, Carl von Ossietzky University Oldenburg, Oldenburg, Lower Saxony, Germany; 3 Federal Institute for Drugs and Medical Devices (BfArM), Kurt-Georg-Kiesinger-Allee, Bonn, Germany; 4 InGef—Institute for Applied Health Research, Berlin, Berlin, Germany; Chiba Daigaku, JAPAN

## Abstract

**Background:**

Antidepressants are frequently used in older patients with depression, but little is known about the comparative safety of individual agents. The objective of the study was to determine the comparative risk of death of antidepressants in older patients with depression.

**Methods and findings:**

We carried out a cohort study from 2004 to 2015 utilizing the German Pharmacoepidemiological Research Database, a population-based database supplied by statutory health insurance providers covering approximately 17% of the general population and all geographical regions. We included 376,846 patients aged 65+ years with a diagnosis of depression who initiated treatment with one of 13 antidepressants (ADs). In total 27,019 patients died during follow-up corresponding to a rate of 119.7 per 1,000 person years. We used proportional hazards models to estimate hazard ratios (HRs) with 95% confidence intervals (CIs) for the risk of death for twelve ADs compared to citalopram. In the primary analysis, we found an increased risk of death associated with the use of amitriptyline (HR 1.15, 95%CI: 1.10–1.20). However, opipramol, trimipramine, doxepin, mirtazapine, fluoxetine, paroxetine, duloxetine, venlafaxine, and St. John’s wort were found to be associated with a lower risk of death. The increased risk of amitriptyline diminished after exclusion of patients with a history of cancer (HR 0.88, 95%CI: 0.82–0.94) and after high-dimensional propensity score (HdPS) adjustment (HR 1.04, 95%CI: 0.95–1.14). In older patients and in those with dementia, differences in risk between most individual ADs and citalopram were smaller. After adjustment by HdPS, the decreased risks for fluoxetine, paroxetine, venlafaxine and mirtazapine compared to citalopram disappeared.

**Conclusions:**

This study suggests that ADs recommended as first-line treatment in patients with depression have a similar safety profile with regard to the risk of death, especially in very old patients and in those with dementia. Further research is needed to investigate the risk of death for individual ADs in specific subgroups such as patients with cancer or cardiovascular disease.

## Introduction

Depression is a common condition in older people with a reported prevalence of 1–4% for major depression and 4–13% for minor depression [1]. Besides non-pharmacological interventions, e.g., cognitive-behavioral therapy, clinical guidelines recommend pharmacological treatment with antidepressants (ADs) [2, 3]. The choice of AD should be guided by the tolerability and safety of the medication as well as the patient’s preference and consideration of side effects such as constipation, sedation, dizziness, and weight gain [4].

Despite their frequent use in older patients with depression [5], the American Geriatric Society Beers Criteria regard the selective serotonin reuptake inhibitor (SSRI) paroxetine and several tricyclic ADs (TCAs) as potentially inappropriate medication in older people due to their anticholinergic and sedating side effects [6]. Moreover, the Screening Tool of Older Persons’ potentially inappropriate Prescriptions (STOPP) criteria recommends avoiding TCAs as first-line therapy in older patients with depression due to a higher risk of adverse drug reactions (ADRs) compared to SSRIs and selective serotonin noradrenaline reuptake inhibitors (SSNRIs) [7]. Because of the effect of TCAs on cardiac conduction and their anticholinergic effects, SSRIs are typically deemed the first-line therapy for the treatment of depression in older people [3, 8], but SSNRIs, mirtazapine and bupropion are also recommended [4].

Although ADRs may increase the risk of death in older patients, observational studies on the comparative safety of antidepressants to support clinicians in their choice of treatment are scarce. A study from the United Kingdom compared the risk of death and other safety outcomes for use of different ADs and AD classes between periods of use and nonuse but did not directly compare individual drugs [9]. Furthermore, ADs which might be frequently used in other European or Asian countries or the United States, e.g., TCAs in general, doxepin and duloxetine [10–12], were not analyzed in the study from the UK. We therefore conducted a large cohort study to directly compare the risk of death in older German patients with depression initiating treatment with 13 different ADs.

## Methods

### Data source

The study was based on claims data (2004–2015) from four German statutory health insurance providers extracted from the German Pharmacoepidemiological Research Database (GePaRD) [13]. The source population consisted of more than 20 million insured persons. Per data year, there is information on approximately 17% of the general population and all geographical regions of Germany are represented. For each person, the database contains demographic information as well as information on hospitalizations, outpatient physician visits, and drug dispensations. The hospital data comprise information on the dates of hospitalization, diagnoses, reasons for admission and discharge, and diagnostic and therapeutic procedures. Claims of outpatient physician visits include outpatient treatments, procedures, and diagnoses. All diagnoses are coded according to the German Modification of the International Statistical Classification of Diseases (ICD-10 GM). Dispensation data are available for all reimbursed outpatient dispensations and include the dates of prescription and dispensation, the amount of substance prescribed, and information on the prescribing physician. Dispensation data are linked via the central pharmaceutical reference number to a pharmaceutical reference database containing information on the anatomical-therapeutic-chemical (ATC) classification code, the defined daily dose (DDD), packaging size, strength, formulation, and the generic and trade name of the respective drugs.

In Germany, the use of health insurance data for scientific research is regulated by the Code of Social Law. All involved statutory health insurance providers as well as the German Federal (Social) Insurance Office and the Senator for Science, Health, and Consumer Protection in Bremen as their responsible authorities approved the use of GePaRD data for this study. Informed consent for studies based on GePaRD is not required by law and according to the Ethics Committee of the University of Bremen these studies are exempt from institutional review board review.

### Study design

We conducted a cohort study in patients who initiated treatment with ADs between January 1, 2005 and December 31, 2015. Patients were included in the cohort if they were continuously insured for at least 365 days before their first AD dispensation (baseline period), had at least one hospital or outpatient diagnosis of depression (ICD-10 GM codes F32, F33, F34.1, F41.2, F43.2) in the baseline period and were 65 years or older at the date of the first AD dispensation (cohort entry). Patients with dispensations of more than one AD at cohort entry were not considered in the analysis. Patients were then followed until either the end of insurance, death, discontinuation or dispensation of another AD indicating switch or combination therapy or end of the study period (December 31, 2015). To account for patients who discontinued therapy due to adverse events shortly before death, these patients were followed for another 30 days.

### Exposure

The exposure status was defined based on the AD dispensation at cohort entry. As information on the intended treatment duration and the prescribed dose is not included in GePaRD, the duration of each dispensation was estimated based on the number of dispensed DDDs, and 150% of the DDD supply was added to each dispensation to account for possible lower doses in older patients [14, 15]. Overlapping dispensations were considered as continuous treatment with subsequent prescriptions. Due to insufficient power, we excluded drugs with fewer than 2,000 patients from the analyses and considered the 13 most commonly prescribed drugs separately in the analyses: TCAs (opipramol, trimipramine, amitriptyline, doxepin), SSRIs (citalopram, escitalopram, sertraline, paroxetine, fluoxetine), SSNRIs (venlafaxine, duloxetine), mirtazapine, and St. John's wort.

### Outcome

Patients were considered dead if the reason for hospital discharge or the reason for deregistration from the insurance was coded as death. A validation study linking data of the Bremen Mortality Index to a subset of the population in GePaRD yielded highly accurate mortality information including the date of death and found that death can be identified validly in GePaRD [16]. Furthermore, it has been shown in a previous study that mortality rates in GePaRD are in good accordance with those from the Federal Statistical Office in Germany indicating that mortality information is adequately captured in GePaRD [17].

### Confounding variables

Possible confounding variables included sex, age at cohort entry, and year of cohort entry. In addition, we considered psychiatric and somatic comorbid conditions including dementia, psychosis, schizophrenia, sleeping disorders, anxiety disorders, Parkinson’s disease, other movement disorders, pain, cancer, diabetes, myocardial infarction, other coronary heart disease, congestive heart failure, atrial fibrillation, ventricular arrhythmia, other cardiac arrhythmias and conduction disorders, valvular disorders, pericardial disorders, peripheral vascular disease, venous thromboembolism and insufficiency, ischemic stroke, other cerebrovascular disease, chronic pulmonary disease, liver disease, renal failure, hypertension, obesity, alcohol abuse and deficiency anemia that may increase the risk of death based on hospital and outpatient diagnoses in the 365 days before cohort entry (S1 Table). Fractures of the lower extremities and surgeries were only considered in the 182 days before cohort entry. Co-medication considered as possible confounding variables including treatment with antidiabetic drugs, insulin, anti-dementia drugs, non-steroidal anti-inflammatory drugs, anti-Parkinson drugs, antipsychotics, antithrombotic drugs, cardiac glycosides, other antihypertensive drugs, vasodilators, beta-adrenergic antagonists, calcium antagonists, ACE inhibitors, angiotensin II antagonists, lipid lowering drugs, glucocorticoids, respiratory drugs, antineoplastic agents and immunosuppressants was ascertained any time prior to cohort entry, whereas hypnotics and sedatives, anxiolytics, and opioids were assessed in the 182 days prior to cohort entry (S2 Table). Furthermore, the number of different drugs used in the baseline period, the percentage of hospitalized person time in the baseline period, and the Charlson comorbidity index [18] as an indicator of overall health status were assessed. Weight loss, fluid and electrolyte disorders, and residence in a nursing home or geriatric care were considered as indicators of frailty.

### Statistical analysis

Crude mortality rates and 95% confidence intervals (CIs) were calculated assuming a Poisson distribution of the events [19]. In the primary analysis, hazard ratios (HRs) and corresponding 95% CIs were estimated using proportional hazards models to compare the risk of death for each individual AD using citalopram as reference since SSRIS are deemed the first-line therapy for depression and citalopram is the most commonly prescribed AD in Germany. Before model building, the assumption of proportional hazards was evaluated by plotting the weighted Schoenfeld residuals against survival times for each independent variable [20]. Age at cohort entry was included as categorical variable in the model to fulfill the assumption of a log-linear relationship of the effect of age on the hazard. Pre-defined subgroup analyses were conducted by age categorized as < 80 years vs. ≥ 80 years and dementia status to identify potential treatment effect modification. Level of significance was 0.05.

To evaluate the robustness of our results, we conducted two sensitivity analyses. First, we excluded patients with a diagnosis of cancer in the baseline period to account for confounding by indication, since several ADs such as amitriptyline, duloxetine, and venlafaxine are also used as analgesics in cancer pain [21]. Second, high-dimensional propensity score (HdPS) adjustment was used as a post-hoc sensitivity analysis to assess the impact of possible unmeasured confounding [22, 23]. The HdPS was defined as the probability of receiving the respective individual AD compared to citalopram depending on a set of up to 500 empirically selected confounding variables derived from in- and outpatient diagnoses, inpatient operations and procedures, and outpatient services and dispensations. In addition, all covariates included in the primary analysis were entered in the HdPS model, and the primary analysis was repeated with additional adjustment for HdPS quintiles after 5% trimming.

All statistical analyses were conducted using SAS 9.3 (SAS Institute Inc., Cary, NC, USA).

## Results

### Characteristics of study cohort

**Fig 1:**
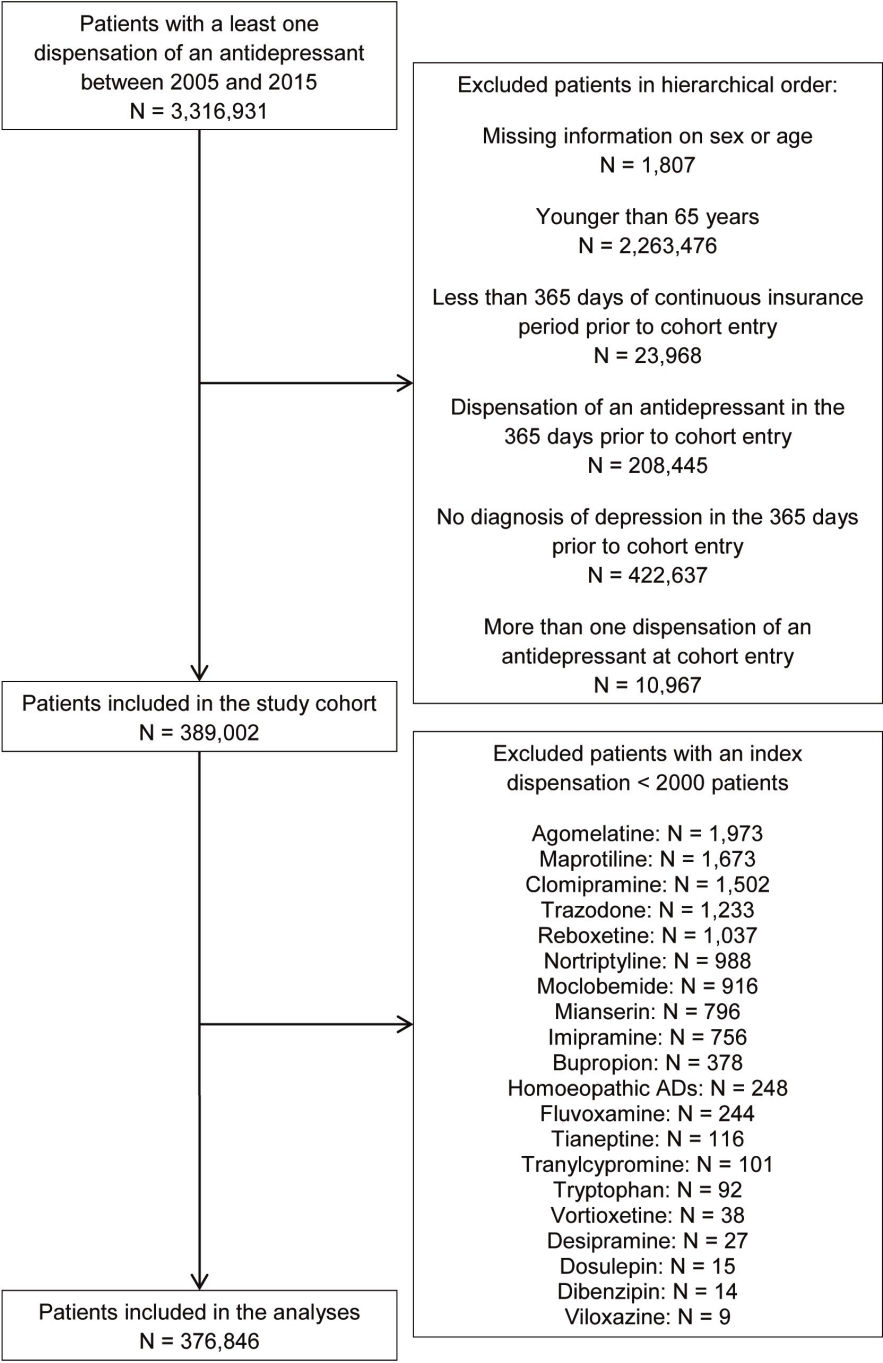
Flow chart of in- and exclusion criteria.

In total, 389,002 patients aged 65 years or older with a diagnosis of depression initiated AD therapy between 2005 and 2015 (Fig 1). The 13 most commonly prescribed ADs included in the analyses comprised 96% of all index AD dispensations (N = 376,846). About 21% of patients started with citalopram, followed by 18% and 16% receiving mirtazapine and amitriptyline, respectively. TCA initiators revealed substantially shorter median follow-up (51–72 days) than all other AD users (98–233 days). The majority of users was female (66–79%). The age distribution was comparable for initiators of all ADs (mean: 73–76 years). Dementia and cardio- and cerebrovascular comorbidities were less often diagnosed in users of St. John's wort and TCAs than in users of all other ADs, with the highest proportion of dementia patients among users of sertraline and citalopram (23% and 22%). A baseline diagnosis of cancer was most common in users of amitriptyline and duloxetine (both 32%), and mirtazapine (31%) and less frequent in patients initiating St. John's wort (26%). The highest proportion of nursing home residents was observed among users of sertraline, citalopram (both 8%), and escitalopram (7%). The highest prevalence of opioid co-medication was found in duloxetine (33%) and amitriptyline users (31%) (S3 and S4 Tables).

### Unadjusted mortality rates by individual antidepressants

The overall mortality rate was 119.7/1,000 person-years. Crude mortality rates were highest for amitriptyline users with 163.3/1,000 person-years (95% CI: 157.7–169.0), followed by mirtazapine and citalopram with 157.1 and 138.9 per 1,000 person-years, respectively (Table 1). The lowest mortality rates were observed for opipramol and St. John's wort with 48.4 and 27.1 per 1,000 person-years, respectively.

**Table 1:** Crude mortality rates per 1,000 person-years by individual antidepressant.

Antidepressant	n	Deaths	Person-years (per 1,000)	Mortality rate per 1,000 person-years (95% CI)
Overall	376,846	27,019	225.81	119.7 (118.2–121.1)
SSRI				
Citalopram	78,422	10,693	76.98	138.9 (136.3–141.6)
Escitalopram	8,868	871	6.59	132.3 (123.6–141.3)
Sertraline	11,897	1,621	13.93	116.4 (110.8–122.2)
Fluoxetine	5,277	322	4.98	64.7 (57.8–72.2)
Paroxetine	4,927	300	5.12	58.6 (52.2–65.6)
SSNRI				
Venlafaxine	8,282	654	6.42	101.8 (94.2–109.9)
Duloxetine	8,043	416	5.20	80.0 (72.5–88.1)
TCA				
Amitriptyline	59,066	3,254	19.93	163.3 (157.7–169.0)
Doxepin	27,837	645	7.24	89.1 (82.3–96.2)
Trimipramine	23,480	343	5.12	66.9 (60.0–74.4)
Opipramol	52,346	794	16.40	48.4 (45.1–51.9)
NASSA				
Mirtazapine	69,714	6,692	42.61	157.1 (153.3–160.9)
Herbal AD				
St. John's wort	18,687	414	15.29	27.1 (24.5–29.8)

Abbreviation: AD, antidepressant; SSRI, selective serotonin reuptake inhibitor; TCA, tricyclic ADs; SSNRI, selective noradrenalin reuptake inhibitor; NASSA, noradrenergic and specific serotonergic Ads, CI, confidence interval

### Adjusted HRs of individual antidepressants vs. citalopram

After covariate adjustment, amitriptyline was associated with a significantly increased risk of death (HR, 1.15; 95% CI, 1.10–1.20) compared to citalopram, whereas a decreased risk of death was seen, in descending order, in users of mirtazapine (HR, 0.94; 95% CI, 0.92–0.97), venlafaxine (HR, 0.92; 95% CI, 0.85–0.99), fluoxetine (HR, 0.86; 95% CI, 0.77–0.96), paroxetine (HR, 0.79; 95% CI, 0.71–0.89), doxepin (HR, 0.79; 95% CI, 0.73–0.86), duloxetine (HR, 0.63; 95% CI, 0.58–0.70), trimipramine (HR, 0.61; 95% CI, 0.55–0.69), opipramol (HR, 0.57; 95% CI, 0.53–0.61), and St. John's wort (HR, 0.42; 95% CI, 0.38–0.47) compared to citalopram (Table 2). There was no significant difference in the risk of death between the use of others ADs and citalopram.

**Table 2:** Hazard ratios for risk of death by individual AD, unadjusted and adjusted for confounders.

	Hazard ratio (95% CI)	
Antidepressant	Unadjusted	Adjusted^a^
Citalopram	1.00	1.00
SSRI		
Escitalopram	0.89 (0.83–0.95), p < .001	0.95 (0.89–1.02), p = 0.137
Sertraline	0.87 (0.83–0.92), p < .001	0.96 (0.91–1.01), p = 0.088
Fluoxetine	0.46 (0.41–0.51), p < .001	0.86 (0.77–0.96), p = 0.006
Paroxetine	0.43 (0.38–0.48), p < .001	0.79 (0.71–0.89), p < .001
SSNRI		
Venlafaxine	0.71 (0.65–0.76), p < .001	0.92 (0.85–0.99), p = 0.032
Duloxetine	0.53 (0.48–0.58), p < .001	0.63 (0.58–0.70), p < .001
TCA		
Amitriptyline	0.89 (0.85–0.92), p < .001	1.15 (1.10–1.20), p < .001
Doxepin	0.45 (0.42–0.49), p < .001	0.79 (0.73–0.86), p < .001
Trimipramine	0.31 (0.28–0.35), p < .001	0.61 (0.55–0.69), p < .001
Opipramol	0.25 (0.23–0.27), p < .001	0.57 (0.53–0.61), p < .001
NASSA		
Mirtazapine	1.01 (0.98–1.04), p = 0.444	0.94 (0.92–0.97), p < .001
Herbal AD		
St. John's wort	0.18 (0.16–0.20), p < .001	0.42 (0.38–0.47), p < .001

Abbreviation: AD, antidepressant; SSRI, selective serotonin reuptake inhibitor; TCA, tricyclic ADs; SSNRI, selective noradrenalin reuptake inhibitor; NASSA, noradrenergic and specific serotonergic ADs, CI, confidence interval

^a^ Hazard ratios were adjusted for female sex, age (categorized), year of index prescription ≥ 2012, dementia, psychosis, schizophrenia, sleeping disorders, anxiety disorders, Parkinson’s disease, other movement disorders, pain, cancer, diabetes, myocardial infarction, other coronary heart disease, congestive heart failure, atrial fibrillation, ventricular arrhythmia, other cardiac arrhythmias and conduction disorders, valvular disorders, pericardial disorders, peripheral vascular disease, venous thromboembolism and insufficiency, ischemic stroke, other cerebrovascular disease, chronic pulmonary disease, liver disease, renal failure, hypertension, obesity, alcohol abuse, fluid and electrolyte disorders, deficiency anemia, any fracture of lower extremities, surgery, weight loss, nursing home residence, insulin, antidiabetic drugs, anti-dementia drugs, opioids, non-steroidal anti-inflammatory drugs, anti-Parkinson drugs, antipsychotics, anxiolytics, hypnotics and sedatives, antithrombotic drugs, cardiac glycosides, other antihypertensive drugs, vasodilators, beta-adrenergic agonists, calcium antagonists, ACE inhibitors, angiotensin II antagonists, lipid lowering drugs, glucocorticoids, respiratory drugs, antineoplastic agents and immunosuppressants, Charlson comorbidity index > 2, hospitalized time > 5%, 5 to 9 drugs, 10 and more drugs.

### Adjusted HRs of individual antidepressants vs. citalopram by subgroups

**Fig 2:**
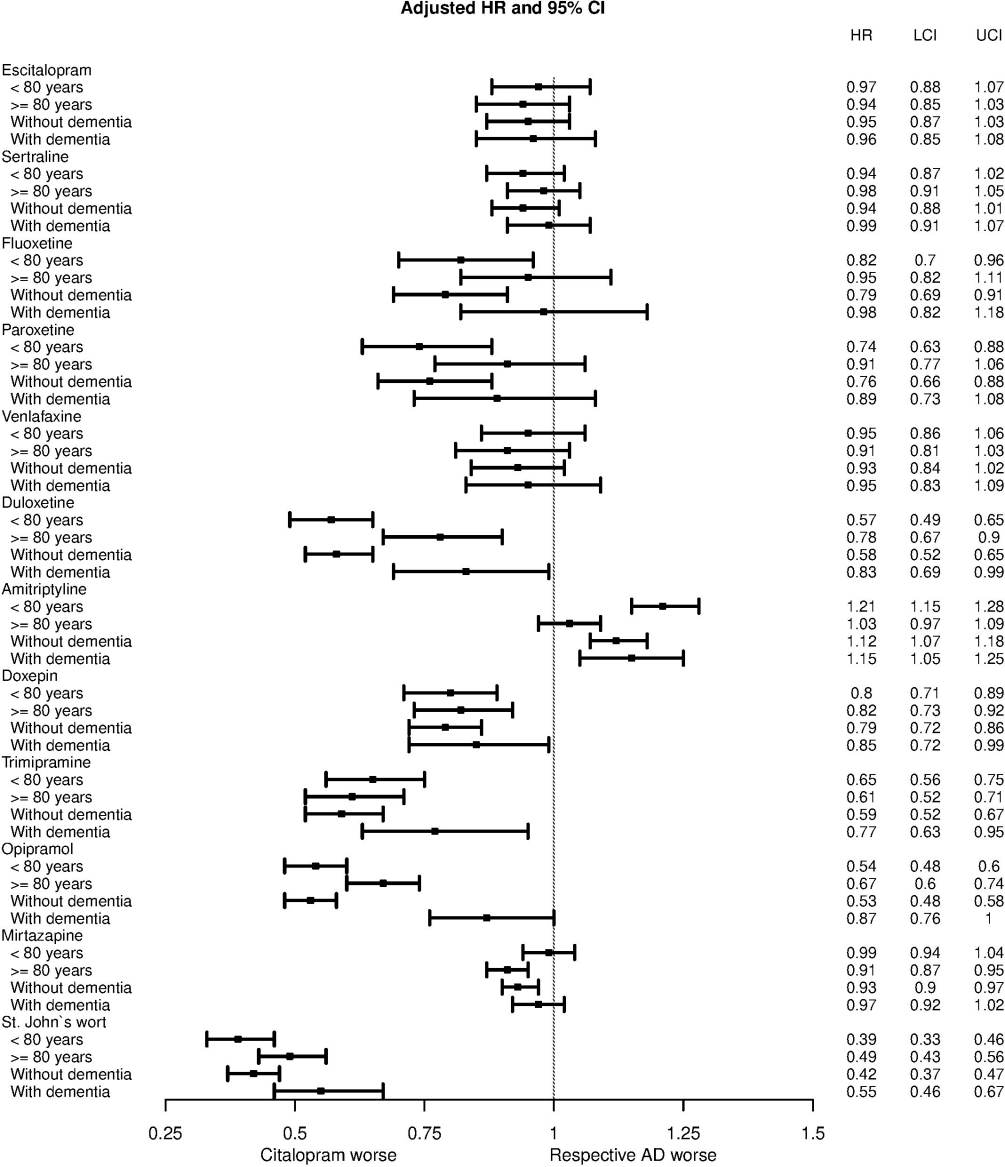
Subgroup analyses by age group and dementia status for adjusted hazard ratios for risk of death.

In patients ≥ 80 years, differences in risk were mostly smaller (fluoxetine, paroxetine, duloxetine) than or similar to those observed for patients younger than 80 years and an increased risk for amitriptyline was only observed in patients younger than 80 years (Fig 2). Only for mirtazapine, a significantly reduced risk was seen in the older group, but not in the younger group. The same pattern was observed for patients with dementia. However, an increased risk of death for amitriptyline was seen both in patients with dementia (HR, 1.15; 95% CI, 1.05–1.25) and patients without dementia (HR, 1.12; 95% CI, 1.07–1.18).

### Sensitivity analyses for adjusted HRs of individual antidepressants vs. citalopram

After the exclusion of cancer patients the increased risk for initiators of amitriptyline disappeared (Table 3). Compared to the primary analysis, exclusion of cancer patients augmented the decreased risk of death for doxepin and trimipramine and attenuated the decreased risk of duloxetine and venlafalxine which was no longer statistically significant After HdPS-adjustment, the difference in the risk of death for fluoxetine, paroxetine, venlafaxine, amitriptyline and mirtazapine vs. citalopram decreased and was no longer statistically significant. The differential risk for duloxetine, doxepin, trimipramine, and opipramol relative to citalopram diminished. For St. John’s wort, this analysis could not be conducted due to a non-overlap of propensity score distributions for St. John’s wort and citalopram.

**Table 3:** Sensitivity analyses of adjusted hazard ratios for risk of death.

		Hazard ratio (95% CI)	
Antidepressant	Primary analysis	Excluding patients with cancer	Adjusted for HdPS
Citalopram	1.00	1.00	1.00
SSRI			
Escitalopram	0.95 (0.89–1.02), p = 0.137	0.94 (0.86–1.03), p = 0.192	0.96 (0.89–1.04), p = 0.356
Sertraline	0.96 (0.91–1.01), p = 0.088	0.98 (0.92–1.04), p = 0.506	1.02 (0.96–1.07), p = 0.575
Fluoxetine	0.86 (0.77–0.96), p = 0.006	0.85 (0.74–0.98), p = 0.028	0.95 (0.84–1.07), p = 0.380
Paroxetine	0.79 (0.71–0.89), p < .001	0.80 (0.69–0.93), p = 0.004	0.90 (0.79–1.03), p = 0.119
SSNRI			
Venlafaxine	0.92 (0.85–0.99), p = 0.032	0.98 (0.89–1.09), p = 0.750	0.99 (0.91–1.08), p = 0.835
Duloxetine	0.63 (0.58–0.70), p < .001	0.72 (0.63–0.83), p < .001	0.83 (0.74–0.92), p < .001
TCA			
Amitriptyline	1.15 (1.10–1.20), p < .001	0.88 (0.82–0.94), p < .001	1.04 (0.95–1.14), p = 0.353
Doxepin	0.79 (0.73–0.86), p < .001	0.68 (0.61–0.77), p < .001	0.87 (0.80–0.95), p = 0.001
Trimipramine	0.61 (0.55–0.69), p < .001	0.54 (0.46–0.64), p < .001	0.69 (0.61–0.77), p < .001
Opipramol	0.57 (0.53–0.61), p < .001	0.57 (0.51–0.63), p < .001	0.71 (0.65–0.76), p < .001
NASSA			
Mirtazapine	0.94 (0.92–0.97), p < .001	0.92 (0.88–0.95), p < .001	0.98 (0.95–1.02), p = 0.335
Herbal AD			
St. John's wort	0.42 (0.38–0.47), p < .001	0.42 (0.37–0.48), p < .001	NA

Abbreviation: AD, antidepressant; SSRI, selective serotonin reuptake inhibitor; TCA, tricyclic ADs; SSNRI, selective noradrenalin reuptake inhibitor; NASSA, noradrenergic and specific serotonergic ADs; HdPS, high-dimensional propensity score; NA, not applicable due to non-overlap of propensity score distributions; CI, confidence interval.

## Discussion and conclusion

In this large observational study, similar safety profiles were observed for several individual antidepressants and citalopram. When compared to citalopram, opipramol, trimipramine, doxepin, mirtazapine, fluoxetine, paroxetine, duloxetine, venlafaxine, and St. John’s wort were associated with a lower risks, but are most likely a result of confounding as differential risks tended toward a null effect in more homogenous subgroups, such as in older patients and in those with dementia, and after additional confounder adjustment by HdPS. The use of amitriptyline was associated with a 15% increased risk of death compared to citalopram, that diminished after exclusion of cancer patients, in patients ≥ 80 years and after additional confounder adjustment by HdPS.

To our knowledge, this is the first study that directly compared the risk of death of several individual ADs to citalopram. Our results are in contrast to those of a large observational study from the UK investigating the association between ADs and death in older patients with depression [9]. Coupland et al. (2011) found a higher risk of death for citalopram (HR, 1.55; 95% CI, 1.48–1.63) than for amitriptyline (HR, 1.10; 95% CI, 1.03–1.18) compared to nonuse of ADs. Furthermore, mirtazapine (HR, 1.76; 95% CI, 1.62–1.91) was associated with one of the highest risks of death. These differences can most likely be explained by different characteristics of the study populations. While the mean age of both study cohorts was comparable, the proportion of female patients included in our study was higher (74% vs. 67%). In addition, the overall mortality rate in our study was twice as high as in the study from the UK, probably due to a higher prevalence of comorbid conditions such as cancer (30% vs. 8%), diabetes (33% vs. 10%), and dementia (13% vs. 1.8%). Unfortunately, the baseline characteristics stratified by individual AD were not reported by Coupland et al. (2011) limiting a direct comparison between both studies [9].

However, we found a similar risk of death in mirtazapine and citalopram users in the subgroup of patients ≥ 80 years which is in line with the findings of an observational study from Sweden that found no increased risk of death in very old antidepressant users, mainly treated with citalopram and mirtazapine, when compared to non-AD users [24].

Furthermore, an observational study based on the Swedish Dementia Registry observed no association of antidepressant use and risk of death in dementia patients [25]. Since the group of AD users consisted of 75% patients treated with citalopram and mirtazapine, the results were mainly driven by these two agents. This is also in line with our results in dementia patients where we found a similar risk of death for mirtazapine and citalopram.

Our study suggests no differential risk of death in users of escitalopram and sertraline vs. citalopram for which consistent results were found in the primary and sensitivity analyses. Excluding cancer patients, the increased risk of death for amitriptyline relative to citalopram disappeared. Prevalence of cancer, pain, and treatment with opioids was one of the highest in amitriptyline users supporting the assumption that amitriptyline is mainly used in patients with depression and a co-indication for pain e.g., due to cancer or neuropathic pain [26]. Consequently, we assume that the observed increased risk of death in the primary analysis might have been related to confounding by indication. In line with this explanation, no differential risk between amitriptyline and citalopram could be observed when using additional adjustment by HdPS and restricting the analysis to patients ≥ 80 years.

Although reduced risks of death for several ADs compared to citalopram were observed in the primary analysis, differences between paroxetine, venlafaxine, mirtazapine and citalopram diminished in our sensitivity analysis using HDPS-adjustment. These findings indicate the beneficial effects observed in the primary analyses may have been related to insufficient control for confounding. This also applies for duloxetine, doxepin, trimipramine and opipramol for which the tendency toward a null effect in the HdPS-adjusted analysis also points to an overestimation of the beneficial effect by unmeasured confounding in the primary analyses. For instance, these agents could have been used in a population with less severe depression or for other indications, a premise supported by the approval of these ADs for further indications. Due to their sedative properties, trimipramine, doxepin, and mirtazapine are also indicated for the treatment of sleeping disorders as reflected by the high proportion of users with this diagnosis in the study cohort [27]. Also, a remarkably high number of duloxetine users had a diagnosis of diabetes and a diagnosis of pain, further supporting this explanation as duloxetine is approved for the treatment of neuropathic pain in diabetes [28]. Furthermore, the high proportion of opipramol and venlafaxine users with coexisting anxiety or sleeping disorders might suggest that these agents were used for the treatment of these indications beside treatment of depression. Similarly, due to the restrictive use of St. John’s Wort for moderate depression [2], the decreased risk relative to citalopram might be explained by confounding by indication. Further, Coupland et al. (2011) found an increasing risk of death for TCAs and SSRIs with higher doses and also observed that TCAs were prescribed at lower doses than SSRIs [9]. If TCAs were also used at lower doses in our study, we might have underestimated the risk for opipramol, trimipramine, doxepin, and amitriptyline.

The strength of this study is its size of about 370,000 new users aged ≥ 65 years offering the possibility to investigate also individual ADs. The huge sample size also provided the opportunity to study vulnerable subgroups such as very old people or patients with dementia. Another strength is the representativeness of the database covering approximatively 17% of the German population. In contrast, a major limitation of observational studies is potential confounding by indication. Although we restricted the cohort to patients with a diagnosis of depression, an effect of confounding by indication on our results cannot be ruled out. In fact, the distribution of comorbidities between antidepressants revealed differences pointing to selective prescribing of specific drugs for co-indications of depression such as dementia, anxiety or sleeping disorders. Furthermore, some possibly important potential confounders, i.e., frailty, are not available in our database and could therefore not be considered in the analysis, but, however, proxy variables as indicators for frailty were considered. To account for possible confounding, we conducted an analysis adjusted by HdPS, and results indicate that the observed differences in the primary analysis might have been attributed to insufficient confounding control. Thus, the still observed beneficial effects of duloxetine, doxepin, trimipramine, opipramol and St. John’s Wort have to be interpreted very cautiously. Although GePaRD contains information on all outpatient dispensations, information on the prescribed daily dose is not available and a dose-adjusted analysis could not be conducted. Furthermore, since information on death certificates is not included in the database, cause of death (e.g. cardiovascular disease or suicide) could not be evaluated.

In conclusion, this study suggests that SSRIs and other ADs recommended by guidelines as first-line treatment in patients with depression have a similar safety profile with regard to the risk of death, especially in patients ≥ 80 years and those with dementia. Although the slightly elevated risk of death observed for amitriptyline is most likely explained by confounding by indication, its use is in general not recommended as first line therapy in elderly patients as per current guidelines and should therefore be avoided except in patients with co-existing indications such as pain. Further research is needed to investigate the risk of death for individual antidepressant after adjustment for dose and to examine the risk of death in specific subgroups, e.g. patients with cancer or cardiovascular disease.

## Supporting information

S1 Table Definition of comorbidities. (DOCX)

S2 Table Definition of comedications. (DOCX)

S3 Table Demographics and baseline characteristics of AD users stratified by individual AD, part 1. (DOCX)

S4 Table Demographics and baseline characteristics of AD users stratified by individual AD, part 2. (DOCX)
