# Supplementary material for: Antidepressants and the risk of death in older patients with depression: A population-based cohort study
Source: PLoS One. 2019 Apr 15;14(4):e0215289. doi: 10.1371/journal.pone.0215289 (PMC6464187; doi:10.1371/journal.pone.0215289)
Supplement: S1 Table — (DOCX) [file pone.0215289.s001.docx]

S1 Table. Definition of comorbidities

| **Confounders** | **ICD-10 GM/OPS/EBM codes** | **Time window** |
| --- | --- | --- |
| Dementia | F00x-F03x, F051, G30x, G311, G3182 | 365 days before cohort entry |
| Schizophrenia | F20x | 365 days before cohort entry |
| Psychoses | F06x, F21x-F29x | 365 days before cohort entry |
| Sleeping disorders | F51x, G47x | 365 days before cohort entry |
| Anxiety disorders | F40x, F41x | 365 days before cohort entry |
| Parkinson`s disease | G20x, G21x, G22 | Any time prior to cohort entry |
| Other movement disorders | F95x, G10, G24x, G25x, G26 | Any time prior to cohort entry |
| Pain | F454x, F6280, G43x, G44x, G50x, G52x-G64, M43x, M45x-M54x, M792x, M81x, M82x, M89x, R51, R52x | Any time prior to cohort entry |
| Cancer, except malignant neoplasm of skin | C00x-C26x, C30x-C34x, C37x-C41x, C43x, C45x-C58x, C60x-C86x, C88x-C97x | Any time prior to cohort entry |
| Diabetes | E10x-E14x | Any time prior to cohort entry |
| Myocardial infarction | I21x | Any time prior to cohort entry |
| Other coronary heart disease | I20x, I24x (excl. I241), I25x (excl. I252 and I255) | Any time prior to cohort entry |
| Congestive heart failure and cardiomyopathy | I099, I255, I42x, I43x, I50x, I971 | Any time prior to cohort entry |
| Atrial fibrillation | I48x | Any time prior to cohort entry |
| Ventricular arrhythmia | I460, I469, I470, I472, I490 | Any time prior to cohort entry |
| Other cardiac arrhythmias and conduction disorders | I44x, I45x, I47x, I49x, R000, R001, R008, T821, Z450, Z950 | Any time prior to cohort entry |
| Valvular disorders (incl. endocarditis) | A520, I011, I05x-I08x, I091, I098, I33x-I39x, Q230-Q233, Z952-Z954 | Any time prior to cohort entry |
| Pericardial disorders | I010, I092, I30x-I32x | Any time prior to cohort entry |
| Peripheral vascular disease | I70x, I71x, I731, I738, I739, I771, I790, I792, K551, K558, K559, Z958x, Z959 | Any time prior to cohort entry |
| Venous thromboembolism and insufficiency | I26x, I80x, I870x, I871, I872 | Any time prior to cohort entry |
| Ischemic stroke and sequelae | I63x, I64, I693, I694 | Any time prior to cohort entry |
| Other cerebrovascular disease | G45x, G46x, H340, I60x-I69x (excl. I63x, I64, I693, I694) | Any time prior to cohort entry |
| Chronic pulmonary disease | J40x-J47x, J60x-J67x, J684, J701, J703 | Any time prior to cohort entry |
| Liver disease | B18x, I850, I864, I982, K70x - K72x, K74x, K76x, Z944 | Any time prior to cohort entry |
| Renal failure | I120, I131, N18x, N19, N250, Z490-Z492, Z940, Z992 | Any time prior to cohort entry |
| Hypertension | I10x-I13x, I15x | Any time prior to cohort entry |
| Obesity | E66x | Any time prior to cohort entry |
| Weight loss | E40-E46, R634, R64 | Any time prior to cohort entry |
| Fluid and electrolyte disorders | E222, E86, E87x | Any time prior to cohort entry |
| Deficiency anemia | D508, D509, D51x-D53x | Any time prior to cohort entry |
| Alcohol abuse | F10x, G312, G621, G721, I426, K292, K70x, K860, T51x, Z502 | Any time prior to cohort entry |
| Senility / nursing home residence | Z74, EBM 03001 or coded hospital discharge to a nursing home | 365 days before cohort entry |
| Any fracture of lower extremities | S72x, S82x, S92x, T023x, T025x, T026x, T120, T121 | 182 days before cohort entry |
| Surgery | OPS: 5x | 182 days before cohort entry |

EBM = Einheitlicher Bewertungsmaßstab (Uniform Value Scale for Outpatient Services), OPS = Operationen- und Prozedurenschlüssel (Operation and Procedure Code)
