# Supplementary material for: Antidepressants and the risk of death in older patients with depression: A population-based cohort study
Source: PLoS One. 2019 Apr 15;14(4):e0215289. doi: 10.1371/journal.pone.0215289 (PMC6464187; doi:10.1371/journal.pone.0215289)
Supplement: S2 Table — (DOCX) [file pone.0215289.s002.docx]

S2 Table. Definition of comedications

| **Confounder** | **ATC code** | **Time window** |
| --- | --- | --- |
| Insulin | A10A | Any time prior to cohort entry |
| Antidiabetic drugs | A10B | Any time prior to cohort entry |
| Anti-dementia drugs | N06D | Any time prior to cohort entry |
| Opioids | N02A | Any time prior to cohort entry |
| Non-steroidal anti-inflammatory drugs | M01A | Any time prior to cohort entry |
| Anti-parkinson drugs | N04 | Any time prior to cohort entry |
| Anxiolytics | N05B | 182 days before cohort entry |
| Hypnotics and sedatives | N05C | 182 days before cohort entry |
| Antithrombotic drugs | B01A | Any time prior to cohort entry |
| Cardiac glycosides | C01A | Any time prior to cohort entry |
| Other antihypertensive drugs | C02 (excl. C02L) | Any time prior to cohort entry |
| Vasodilators | C01D, C04A, C04B | Any time prior to cohort entry |
| Beta-adrenergic agonists | C07 | Any time prior to cohort entry |
| Calcium antagonists | C08, | Any time prior to cohort entry |
| ACE inhibitors | C09A, C09B | Any time prior to cohort entry |
| Angiotensin II antagonists | C09C, C09D | Any time prior to cohort entry |
| Lipid lowering drugs | C10 | Any time prior to cohort entry |
| Glucocorticoids | H02AB | Any time prior to cohort entry |
| Respiratory drugs | R03 | Any time prior to cohort entry |
| Antineoplastic agents and immunosuppressants | L01, L04 | Any time prior to cohort entry |
