# Supplementary material for: Antidepressants and the risk of death in older patients with depression: A population-based cohort study
Source: PLoS One. 2019 Apr 15;14(4):e0215289. doi: 10.1371/journal.pone.0215289 (PMC6464187; doi:10.1371/journal.pone.0215289)
Supplement: S3 Table — (DOCX) [file pone.0215289.s003.docx]

S3 Table. Demographics and baseline characteristics of AD users stratified by individual AD, part 1

|  | **Amitriptyline**  **N = 59,066** | **Opipramol**  **N = 52,346** | **Doxepin**  **N = 27,837** | **Trimipramine**  **N = 23,480** | **Mirtazapine**  **N = 69,714** | **St. John's wort**  **N = 18,687** |
| --- | --- | --- | --- | --- | --- | --- |
| Female sex | 45,682 (77.3%) | 41,513 (79.3%) | 21,114 (75.8%) | 18,079 (77.0%) | 49,563 (71.1%) | 14,477 (77.5%) |
| Age at cohort entry, mean (SD), years | 73.8 (6.9) | 72.6 (6.5) | 73.5 (6.9) | 73.2 (6.9) | 75.5 (7.6) | 73.2 (6.8) |
| Follow-up, median (Q1 – Q3), days | 72 (49-114) | 72 (55-114) | 60 (43-93) | 51 (50-72) | 98 (69-221) | 233 (135-368) |
| **Comorbidities** |  |  |  |  |  |  |
| Dementia^b^ | 4,757 ( 8.1%) | 3,099 ( 5.9%) | 2,394 ( 8.6%) | 1,886 ( 8.0%) | 12,311 (17.7%) | 1,339 ( 7.2%) |
| Psychoses^b^ | 2,295 ( 3.9%) | 1,607 ( 3.1%) | 1,402 ( 5.0%) | 1,039 ( 4.4%) | 5,005 ( 7.2%) | 526 ( 2.8%) |
| Schizophrenia^b^ | 271 ( 0.5%) | 236 ( 0.5%) | 182 ( 0.7%) | 184 ( 0.8%) | 800 ( 1.1%) | 50 ( 0.3%) |
| Sleeping disorders^b^ | 14,465 (24.5%) | 13,017 (24.9%) | 8,160 (29.3%) | 8,633 (36.8%) | 20,619 (29.6%) | 3,486 (18.7%) |
| Anxiety disorders^b^ | 8,884 (15.0%) | 13,021 (24.9%) | 5,321 (19.1%) | 4,498 (19.2%) | 12,242 (17.6%) | 2,539 (13.6%) |
| Parkinson`s disease^a^ | 2,360 ( 4.0%) | 1,610 ( 3.1%) | 1,074 ( 3.9%) | 1,081 ( 4.6%) | 4,518 ( 6.5%) | 491 ( 2.6%) |
| Other movement disorders^a^ | 6,781 (11.5%) | 5,265 (10.1%) | 2,639 ( 9.5%) | 2,823 (12.0%) | 7,683 (11.0%) | 1,580 ( 8.5%) |
| Alcohol abuse^a^ | 2,456 ( 4.2%) | 1,854 ( 3.5%) | 1,769 ( 6.4%) | 1,015 ( 4.3%) | 4,332 ( 6.2%) | 548 ( 2.9%) |
| Pain^a^ | 55,726 (94.3%) | 48,043 (91.8%) | 25,036 (89.9%) | 21,568 (91.9%) | 63,438 (91.0%) | 17,037 (91.2%) |
| Cancer, except malignant neoplasm of skin^a^ | 19,013 (32.2%) | 14,272 (27.3%) | 7,464 (26.8%) | 6,532 (27.8%) | 21,718 (31.2%) | 4,825 (25.8%) |
| Diabetes^a^ | 19,062 (32.3%) | 14,720 (28.1%) | 8,352 (30.0%) | 6,892 (29.4%) | 23,427 (33.6%) | 4,839 (25.9%) |
| Acute myocardial infarction^a^ | 3,593 ( 6.1%) | 2,701 ( 5.2%) | 1,629 ( 5.9%) | 1,277 ( 5.4%) | 5,793 ( 8.3%) | 785 ( 4.2%) |
| Other coronary heart disease^a^ | 24,473 (41.4%) | 20,614 (39.4%) | 11,087 (39.8%) | 9,425 (40.1%) | 31,653 (45.4%) | 6,753 (36.1%) |
| Congestive heart failure and cardiomyopathy^a^ | 17,100 (29.0%) | 11,759 (22.5%) | 7,251 (26.0%) | 5,764 (24.5%) | 24,470 (35.1%) | 3,952 (21.1%) |
| Atrial fibrillation^a^ | 8,644 (14.6%) | 6,620 (12.6%) | 3,882 (13.9%) | 3,175 (13.5%) | 14,898 (21.4%) | 2,067 (11.1%) |
| Ventricular arrhythmia^a^ | 1,079 ( 1.8%) | 917 ( 1.8%) | 489 ( 1.8%) | 386 ( 1.6%) | 1,961 ( 2.8%) | 249 ( 1.3%) |
| Other cardiac arrhythmias and conduction disorders^a^ | 23,485 (39.8%) | 21,318 (40.7%) | 10,815 (38.9%) | 9,297 (39.6%) | 31,097 (44.6%) | 7,119 (38.1%) |
| Valvular disorders (incl. endocarditis)^a^ | 12,317 (20.9%) | 10,482 (20.0%) | 5,279 (19.0%) | 4,705 (20.0%) | 17,523 (25.1%) | 3,541 (18.9%) |
| Pericardial disorders^a^ | 765 ( 1.3%) | 592 ( 1.1%) | 281 ( 1.0%) | 261 ( 1.1%) | 1,033 ( 1.5%) | 199 ( 1.1%) |
| Peripheral vascular disease^a^ | 18,095 (30.6%) | 13,592 (26.0%) | 7,271 (26.1%) | 6,232 (26.5%) | 22,967 (32.9%) | 4,584 (24.5%) |
| Venous thromboembolism and insufficiency | 15,472 (26.2%) | 12,503 (23.9%) | 6,077 (21.8%) | 5,489 (23.4%) | 17,496 (25.1%) | 4,393 (23.5%) |
| Ischemic stroke and sequelae | 6,228 (10.5%) | 4,071 ( 7.8%) | 2,572 ( 9.2%) | 2,128 ( 9.1%) | 10,989 (15.8%) | 1,530 ( 8.2%) |
| Other cerebrovascular disease^a^ | 18,790 (31.8%) | 15,781 (30.1%) | 8,389 (30.1%) | 7,248 (30.9%) | 26,149 (37.5%) | 5,510 (29.5%) |
| Hypertension^a^ | 48,343 (81.8%) | 42,348 (80.9%) | 22,567 (81.1%) | 18,820 (80.2%) | 59,270 (85.0%) | 14,432 (77.2%) |
| Chronic pulmonary disease^a^ | 28,250 (47.8%) | 24,275 (46.4%) | 12,445 (44.7%) | 10,736 (45.7%) | 32,703 (46.9%) | 8,133 (43.5%) |
| Liver disease^a^ | 16,005 (27.1%) | 13,711 (26.2%) | 7,330 (26.3%) | 6,024 (25.7%) | 18,981 (27.2%) | 4,439 (23.8%) |
| Renal failure^a^ | 10,806 (18.3%) | 7,005 (13.4%) | 4,358 (15.7%) | 3,485 (14.8%) | 16,181 (23.2%) | 2,148 (11.5%) |
| Obesity^a^ | 17,222 (29.2%) | 13,675 (26.1%) | 7,149 (25.7%) | 6,176 (26.3%) | 18,342 (26.3%) | 4,209 (22.5%) |
| Any fracture of lower extremities^c^ | 1,023 ( 1.7%) | 562 ( 1.1%) | 387 ( 1.4%) | 338 ( 1.4%) | 1,583 ( 2.3%) | 210 ( 1.1%) |
| Surgery^c^ | 12,208 (20.7%) | 7,133 (13.6%) | 4,227 (15.2%) | 3,500 (14.9%) | 15,336 (22.0%) | 2,211 (11.8%) |
| Fluid and electrolyte disorders^a^ | 14,419 (24.4%) | 8,685 (16.6%) | 5,598 (20.1%) | 4,345 (18.5%) | 23,738 (34.1%) | 2,788 (14.9%) |
| Deficiency anemia^a^ | 6,390 (10.8%) | 4,251 ( 8.1%) | 2,549 ( 9.2%) | 2,058 ( 8.8%) | 8,608 (12.3%) | 1,491 ( 8.0%) |
| Weight loss^a^ | 5,281 ( 8.9%) | 3,402 ( 6.5%) | 2,022 ( 7.3%) | 1,666 ( 7.1%) | 8,994 (12.9%) | 1,171 ( 6.3%) |
| Nursing home residence^b^ | 1,918 ( 3.2%) | 735 ( 1.4%) | 783 ( 2.8%) | 553 ( 2.4%) | 4,894 ( 7.0%) | 245 ( 1.3%) |
| Charlson Comorbidity Index > 2^a^ | 34,131 (57.8%) | 25,509 (48.7%) | 14,129 (50.8%) | 11,859 (50.5%) | 43,517 (62.4%) | 8,481 (45.4%) |
| Hospitalized time > 5%^b^ | 12,925 (21.9%) | 4,734 ( 9.0%) | 3,894 (14.0%) | 2,676 (11.4%) | 21,902 (31.4%) | 1,328 ( 7.1%) |
| **Comedication** |  |  |  |  |  |  |
| Anti-dementia drugs^a^ | 2,042 ( 3.5%) | 1,775 ( 3.4%) | 1,047 ( 3.8%) | 967 ( 4.1%) | 4,580 ( 6.6%) | 946 ( 5.1%) |
| Anti-parkinson drugs^a^ | 5,064 ( 8.6%) | 3,218 ( 6.1%) | 1,919 ( 6.9%) | 2,194 ( 9.3%) | 6,574 ( 9.4%) | 886 ( 4.7%) |
| Antipsychotics^a^ | 8,022 (13.6%) | 6,912 (13.2%) | 4,452 (16.0%) | 3,783 (16.1%) | 14,228 (20.4%) | 1,653 ( 8.8%) |
| Anxiolytics^c^ | 7,288 (12.3%) | 7,140 (13.6%) | 4,173 (15.0%) | 3,234 (13.8%) | 11,167 (16.0%) | 1,704 ( 9.1%) |
| Hypnotics and sedatives^c^ | 5,946 (10.1%) | 4,830 ( 9.2%) | 3,170 (11.4%) | 3,147 (13.4%) | 9,348 (13.4%) | 1,309 ( 7.0%) |
| Opioids^c^ | 18,162 (30.7%) | 5,712 (10.9%) | 4,551 (16.3%) | 3,524 (15.0%) | 13,033 (18.7%) | 1,730 ( 9.3%) |
| Non-steroidal anti-inflammatory drugs^a^ | 48,000 (81.3%) | 39,984 (76.4%) | 20,552 (73.8%) | 18,029 (76.8%) | 53,439 (76.7%) | 13,458 (72.0%) |
| Insulin^a^ | 3,836 ( 6.5%) | 2,193 ( 4.2%) | 1,405 ( 5.0%) | 1,071 ( 4.6%) | 4,509 ( 6.5%) | 645 ( 3.5%) |
| Antidiabetic drugs^a^ | 8,013 (13.6%) | 5,811 (11.1%) | 3,345 (12.0%) | 2,739 (11.7%) | 9,772 (14.0%) | 1,832 ( 9.8%) |
| Antithrombotic drugs^a^ | 24,145 (40.9%) | 18,608 (35.5%) | 10,152 (36.5%) | 8,662 (36.9%) | 32,818 (47.1%) | 6,264 (33.5%) |
| Cardiac glycosides^a^ | 4,555 ( 7.7%) | 3,204 ( 6.1%) | 2,181 ( 7.8%) | 1,626 ( 6.9%) | 7,011 (10.1%) | 1,123 ( 6.0%) |
| Other antihypertensive drugs^a^ | 3,976 ( 6.7%) | 3,535 ( 6.8%) | 1,836 ( 6.6%) | 1,510 ( 6.4%) | 5,378 ( 7.7%) | 929 ( 5.0%) |
| Vasodilators^a^ | 14,894 (25.2%) | 12,503 (23.9%) | 6,625 (23.8%) | 5,699 (24.3%) | 18,935 (27.2%) | 3,990 (21.4%) |
| Beta-adrenergic agonists^a^ | 30,638 (51.9%) | 27,628 (52.8%) | 14,598 (52.4%) | 12,191 (51.9%) | 39,153 (56.2%) | 8,490 (45.4%) |
| Calcium antagonists^a^ | 19,422 (32.9%) | 16,734 (32.0%) | 8,816 (31.7%) | 7,451 (31.7%) | 25,431 (36.5%) | 5,140 (27.5%) |
| ACE inhibitors^a^ | 28,444 (48.2%) | 23,713 (45.3%) | 13,019 (46.8%) | 10,660 (45.4%) | 37,166 (53.3%) | 7,662 (41.0%) |
| Angiotensin II antagonists^a^ | 13,864 (23.5%) | 13,056 (24.9%) | 6,038 (21.7%) | 5,555 (23.7%) | 17,917 (25.7%) | 4,254 (22.8%) |
| Lipid lowering drugs^a^ | 20,899 (35.4%) | 18,646 (35.6%) | 9,330 (33.5%) | 8,466 (36.1%) | 26,968 (38.7%) | 5,877 (31.4%) |
| Glucocorticoids^a^ | 22,067 (37.4%) | 15,950 (30.5%) | 8,173 (29.4%) | 7,390 (31.5%) | 22,901 (32.8%) | 4,811 (25.7%) |
| Respiratory drugs^a^ | 16,748 (28.4%) | 14,145 (27.0%) | 7,123 (25.6%) | 6,254 (26.6%) | 19,235 (27.6%) | 4,410 (23.6%) |
| Antineoplastic agents and immunosuppressants^a^ | 3,392 ( 5.7%) | 1,534 ( 2.9%) | 927 ( 3.3%) | 797 ( 3.4%) | 3,208 ( 4.6%) | 620 ( 3.3%) |
| 1 to 4 drugs^c^ | 15,811 (26.8%) | 19,561 (37.4%) | 9,654 (34.7%) | 8,182 (34.8%) | 19,898 (28.5%) | 8,290 (44.4%) |
| 5 to 9 drugs^c^ | 20,051 (33.9%) | 18,446 (35.2%) | 9,513 (34.2%) | 8,148 (34.7%) | 23,480 (33.7%) | 5,999 (32.1%) |
| 10 and more drugs^c^ | 21,649 (36.7%) | 12,323 (23.5%) | 7,602 (27.3%) | 6,323 (26.9%) | 23,818 (34.2%) | 3,293 (17.6%) |

^a^ Assessed any time prior to cohort entry.

^b^ Assessed in the 365 days before cohort entry.

^c^ Assessed in the 182 days before cohort entry.
