# Supplementary material for: Antidepressants and the risk of death in older patients with depression: A population-based cohort study
Source: PLoS One. 2019 Apr 15;14(4):e0215289. doi: 10.1371/journal.pone.0215289 (PMC6464187; doi:10.1371/journal.pone.0215289)
Supplement: S4 Table — (DOCX) [file pone.0215289.s004.docx]

S4 Table. Demographics and baseline characteristics of AD users stratified by individual AD, part 2

|  | **Citalopram**  **N = 78,422** | **Sertraline**  **N = 11,897** | **Escitalopram**  **N = 8,868** | **Fluoxetine**  **N = 5,277** | **Paroxetine**  **N = 4,927** | **Venlafaxine**  **N = 8,282** | **Duloxetine**  **N = 8,043** |
| --- | --- | --- | --- | --- | --- | --- | --- |
| Female sex | 54,010 (68.9%) | 8,048 (67.6%) | 5,891 (66.4%) | 3,751 (71.1%) | 3,629 (73.7%) | 5,643 (68.1%) | 5,492 (68.3%) |
| Age at cohort entry, mean (SD), years | 75.9 (7.7) | 75.1 (7.6) | 74.8 (7.5) | 72.7 (6.9) | 72.8 (6.8) | 73.5 (7.0) | 73.6 (6.8) |
| Follow-up, median (Q1 – Q3), days | 155 (85-392) | 232 (97-500) | 155 (80-326) | 155 (80-341) | 155 (80-385) | 124 (66-274) | 100 (65-260) |
| **Comorbidities** |  |  |  |  |  |  |  |
| Dementia^b^ | 16,835 (21.5%) | 2,680 (22.5%) | 1,741 (19.6%) | 638 (12.1%) | 585 (11.9%) | 1,309 (15.8%) | 937 (11.6%) |
| Psychoses^b^ | 5,750 ( 7.3%) | 1,029 ( 8.6%) | 744 ( 8.4%) | 296 ( 5.6%) | 285 ( 5.8%) | 691 ( 8.3%) | 487 ( 6.1%) |
| Schizophrenia^b^ | 838 ( 1.1%) | 163 ( 1.4%) | 124 ( 1.4%) | 51 ( 1.0%) | 55 ( 1.1%) | 127 ( 1.5%) | 78 ( 1.0%) |
| Sleeping disorders^b^ | 14,888 (19.0%) | 2,244 (18.9%) | 1,758 (19.8%) | 1,055 (20.0%) | 951 (19.3%) | 1,774 (21.4%) | 1,795 (22.3%) |
| Anxiety disorders^b^ | 11,605 (14.8%) | 2,068 (17.4%) | 1,686 (19.0%) | 854 (16.2%) | 1,499 (30.4%) | 1,866 (22.5%) | 1,372 (17.1%) |
| Parkinson`s disease^a^ | 5,784 ( 7.4%) | 985 ( 8.3%) | 721 ( 8.1%) | 266 ( 5.0%) | 264 ( 5.4%) | 564 ( 6.8%) | 564 ( 7.0%) |
| Other movement disorders^a^ | 7,450 ( 9.5%) | 1,160 ( 9.8%) | 971 (10.9%) | 492 ( 9.3%) | 495 (10.0%) | 869 (10.5%) | 1,315 (16.3%) |
| Alcohol abuse^a^ | 4,427 ( 5.6%) | 731 ( 6.1%) | 510 ( 5.8%) | 248 ( 4.7%) | 210 ( 4.3%) | 525 ( 6.3%) | 452 ( 5.6%) |
| Pain^a^ | 70,404 (89.8%) | 10,477 (88.1%) | 7,891 (89.0%) | 4,686 (88.8%) | 4,424 (89.8%) | 7,443 (89.9%) | 7,735 (96.2%) |
| Cancer, except malignant neoplasm of skin^a^ | 23,035 (29.4%) | 3,382 (28.4%) | 2,600 (29.3%) | 1,407 (26.7%) | 1,310 (26.6%) | 2,413 (29.1%) | 2,595 (32.3%) |
| Diabetes^a^ | 28,436 (36.3%) | 4,279 (36.0%) | 3,064 (34.6%) | 1,617 (30.6%) | 1,468 (29.8%) | 2,793 (33.7%) | 3,453 (42.9%) |
| Acute myocardial infarction^a^ | 6,583 ( 8.4%) | 1,038 ( 8.7%) | 760 ( 8.6%) | 313 ( 5.9%) | 306 ( 6.2%) | 553 ( 6.7%) | 633 ( 7.9%) |
| Other coronary heart disease^a^ | 35,374 (45.1%) | 5,403 (45.4%) | 4,064 (45.8%) | 2,027 (38.4%) | 1,967 (39.9%) | 3,443 (41.6%) | 3,798 (47.2%) |
| Congestive heart failure and cardiomyopathy^a^ | 28,373 (36.2%) | 4,171 (35.1%) | 3,110 (35.1%) | 1,337 (25.3%) | 1,225 (24.9%) | 2,363 (28.5%) | 2,679 (33.3%) |
| Atrial fibrillation^a^ | 17,391 (22.2%) | 2,554 (21.5%) | 1,954 (22.0%) | 730 (13.8%) | 688 (14.0%) | 1,500 (18.1%) | 1,417 (17.6%) |
| Ventricular arrhythmia^a^ | 2,221 ( 2.8%) | 351 ( 3.0%) | 266 ( 3.0%) | 106 ( 2.0%) | 102 ( 2.1%) | 202 ( 2.4%) | 213 ( 2.6%) |
| Other cardiac arrhythmias and conduction disorders^a^ | 34,153 (43.6%) | 4,988 (41.9%) | 4,004 (45.2%) | 1,877 (35.6%) | 1,884 (38.2%) | 3,426 (41.4%) | 3,610 (44.9%) |
| Valvular disorders (incl. endocarditis)^a^ | 19,083 (24.3%) | 2,789 (23.4%) | 2,220 (25.0%) | 953 (18.1%) | 940 (19.1%) | 1,838 (22.2%) | 1,970 (24.5%) |
| Pericardial disorders^a^ | 1,057 ( 1.3%) | 175 ( 1.5%) | 130 ( 1.5%) | 58 ( 1.1%) | 49 ( 1.0%) | 111 ( 1.3%) | 135 ( 1.7%) |
| Peripheral vascular disease^a^ | 25,853 (33.0%) | 3,774 (31.7%) | 2,835 (32.0%) | 1,413 (26.8%) | 1,265 (25.7%) | 2,422 (29.2%) | 2,950 (36.7%) |
| Venous thromboembolism and insufficiency | 19,830 (25.3%) | 2,878 (24.2%) | 2,197 (24.8%) | 1,119 (21.2%) | 1,107 (22.5%) | 2,001 (24.2%) | 2,348 (29.2%) |
| Ischemic stroke and sequelae | 15,326 (19.5%) | 2,266 (19.0%) | 1,852 (20.9%) | 693 (13.1%) | 561 (11.4%) | 1,271 (15.3%) | 1,153 (14.3%) |
| Other cerebrovascular disease^a^ | 31,593 (40.3%) | 4,730 (39.8%) | 3,702 (41.7%) | 1,668 (31.6%) | 1,642 (33.3%) | 2,994 (36.2%) | 3,133 (39.0%) |
| Hypertension^a^ | 67,140 (85.6%) | 10,044 (84.4%) | 7,436 (83.9%) | 4,143 (78.5%) | 3,884 (78.8%) | 6,800 (82.1%) | 6,926 (86.1%) |
| Chronic pulmonary disease^a^ | 36,323 (46.3%) | 5,317 (44.7%) | 3,947 (44.5%) | 2,350 (44.5%) | 2,087 (42.4%) | 3,748 (45.3%) | 4,070 (50.6%) |
| Liver disease^a^ | 20,787 (26.5%) | 3,117 (26.2%) | 2,381 (26.8%) | 1,322 (25.1%) | 1,273 (25.8%) | 2,197 (26.5%) | 2,463 (30.6%) |
| Renal failure^a^ | 18,855 (24.0%) | 2,856 (24.0%) | 2,090 (23.6%) | 786 (14.9%) | 725 (14.7%) | 1,616 (19.5%) | 1,964 (24.4%) |
| Obesity^a^ | 21,879 (27.9%) | 3,384 (28.4%) | 2,434 (27.4%) | 1,497 (28.4%) | 1,292 (26.2%) | 2,373 (28.7%) | 2,917 (36.3%) |
| Any fracture of lower extremities^c^ | 1,799 ( 2.3%) | 279 ( 2.3%) | 206 ( 2.3%) | 60 ( 1.1%) | 72 ( 1.5%) | 162 ( 2.0%) | 147 ( 1.8%) |
| Surgery^c^ | 16,243 (20.7%) | 2,403 (20.2%) | 2,014 (22.7%) | 763 (14.5%) | 715 (14.5%) | 1,642 (19.8%) | 1,674 (20.8%) |
| Fluid and electrolyte disorders^a^ | 26,664 (34.0%) | 3,916 (32.9%) | 3,001 (33.8%) | 1,031 (19.5%) | 1,003 (20.4%) | 2,313 (27.9%) | 2,370 (29.5%) |
| Deficiency anemia^a^ | 9,745 (12.4%) | 1,395 (11.7%) | 1,056 (11.9%) | 442 ( 8.4%) | 432 ( 8.8%) | 853 (10.3%) | 1,079 (13.4%) |
| Weight loss^a^ | 8,688 (11.1%) | 1,233 (10.4%) | 983 (11.1%) | 377 ( 7.1%) | 377 ( 7.7%) | 920 (11.1%) | 768 ( 9.5%) |
| Nursing home residence^b^ | 6,060 ( 7.7%) | 913 ( 7.7%) | 648 ( 7.3%) | 219 ( 4.2%) | 165 ( 3.3%) | 433 ( 5.2%) | 352 ( 4.4%) |
| Charlson Comorbidity Index > 2^a^ | 50,021 (63.8%) | 7,374 (62.0%) | 5,619 (63.4%) | 2,745 (52.0%) | 2,436 (49.4%) | 4,790 (57.8%) | 5,277 (65.6%) |
| Hospitalized time > 5%^b^ | 22,570 (28.8%) | 3,614 (30.4%) | 3,226 (36.4%) | 769 (14.6%) | 750 (15.2%) | 2,398 (29.0%) | 2,363 (29.4%) |
| **Comedication** |  |  |  |  |  |  |  |
| Anti-dementia drugs^a^ | 6,596 ( 8.4%) | 1,048 ( 8.8%) | 760 ( 8.6%) | 277 ( 5.2%) | 273 ( 5.5%) | 577 ( 7.0%) | 454 ( 5.6%) |
| Anti-parkinson drugs^a^ | 7,308 ( 9.3%) | 1,202 (10.1%) | 931 (10.5%) | 446 ( 8.5%) | 390 ( 7.9%) | 813 ( 9.8%) | 1,189 (14.8%) |
| Antipsychotics^a^ | 14,580 (18.6%) | 2,513 (21.1%) | 1,778 (20.0%) | 887 (16.8%) | 900 (18.3%) | 1,886 (22.8%) | 1,450 (18.0%) |
| Anxiolytics^c^ | 9,039 (11.5%) | 1,440 (12.1%) | 1,170 (13.2%) | 659 (12.5%) | 781 (15.9%) | 1,142 (13.8%) | 947 (11.8%) |
| Hypnotics and sedatives^c^ | 6,781 ( 8.6%) | 1,007 ( 8.5%) | 867 ( 9.8%) | 469 ( 8.9%) | 460 ( 9.3%) | 727 ( 8.8%) | 730 ( 9.1%) |
| Opioids^c^ | 12,846 (16.4%) | 1,776 (14.9%) | 1,332 (15.0%) | 708 (13.4%) | 648 (13.2%) | 1,243 (15.0%) | 2,666 (33.1%) |
| Non-steroidal anti-inflammatory drugs^a^ | 59,135 (75.4%) | 8,553 (71.9%) | 6,497 (73.3%) | 3,786 (71.7%) | 3,539 (71.8%) | 6,213 (75.0%) | 6,852 (85.2%) |
| Insulin^a^ | 6,316 ( 8.1%) | 969 ( 8.1%) | 666 ( 7.5%) | 293 ( 5.6%) | 276 ( 5.6%) | 564 ( 6.8%) | 998 (12.4%) |
| Antidiabetic drugs^a^ | 12,890 (16.4%) | 1,925 (16.2%) | 1,365 (15.4%) | 721 (13.7%) | 625 (12.7%) | 1,192 (14.4%) | 1,793 (22.3%) |
| Antithrombotic drugs^a^ | 37,920 (48.4%) | 5,548 (46.6%) | 4,207 (47.4%) | 1,915 (36.3%) | 1,826 (37.1%) | 3,512 (42.4%) | 3,981 (49.5%) |
| Cardiac glycosides^a^ | 8,565 (10.9%) | 1,213 (10.2%) | 919 (10.4%) | 392 ( 7.4%) | 347 ( 7.0%) | 671 ( 8.1%) | 718 ( 8.9%) |
| Other antihypertensive drugs^a^ | 5,757 ( 7.3%) | 902 ( 7.6%) | 647 ( 7.3%) | 324 ( 6.1%) | 307 ( 6.2%) | 560 ( 6.8%) | 745 ( 9.3%) |
| Vasodilators^a^ | 20,370 (26.0%) | 3,060 (25.7%) | 2,291 (25.8%) | 1,163 (22.0%) | 1,147 (23.3%) | 1,941 (23.4%) | 2,387 (29.7%) |
| Beta-adrenergic agonists^a^ | 43,455 (55.4%) | 6,468 (54.4%) | 4,862 (54.8%) | 2,523 (47.8%) | 2,485 (50.4%) | 4,388 (53.0%) | 4,518 (56.2%) |
| Calcium antagonists^a^ | 28,209 (36.0%) | 4,194 (35.3%) | 3,079 (34.7%) | 1,544 (29.3%) | 1,501 (30.5%) | 2,659 (32.1%) | 3,009 (37.4%) |
| ACE inhibitors^a^ | 42,944 (54.8%) | 6,216 (52.2%) | 4,522 (51.0%) | 2,329 (44.1%) | 2,152 (43.7%) | 3,986 (48.1%) | 4,150 (51.6%) |
| Angiotensin II antagonists^a^ | 19,041 (24.3%) | 2,945 (24.8%) | 2,451 (27.6%) | 1,142 (21.6%) | 1,196 (24.3%) | 2,176 (26.3%) | 2,559 (31.8%) |
| Lipid lowering drugs^a^ | 30,704 (39.2%) | 4,621 (38.8%) | 3,396 (38.3%) | 1,776 (33.7%) | 1,719 (34.9%) | 3,162 (38.2%) | 3,431 (42.7%) |
| Glucocorticoids^a^ | 23,905 (30.5%) | 3,379 (28.4%) | 2,754 (31.1%) | 1,441 (27.3%) | 1,398 (28.4%) | 2,602 (31.4%) | 3,206 (39.9%) |
| Respiratory drugs^a^ | 20,555 (26.2%) | 3,069 (25.8%) | 2,277 (25.7%) | 1,378 (26.1%) | 1,193 (24.2%) | 2,111 (25.5%) | 2,482 (30.9%) |
| Antineoplastic agents and immunosuppressants^a^ | 3,193 ( 4.1%) | 450 ( 3.8%) | 411 ( 4.6%) | 151 ( 2.9%) | 150 ( 3.0%) | 331 ( 4.0%) | 434 ( 5.4%) |
| 1 to 4 drugs^c^ | 23,474 (29.9%) | 3,620 (30.4%) | 2,741 (30.9%) | 1,917 (36.3%) | 1,785 (36.2%) | 2,808 (33.9%) | 1,684 (20.9%) |
| 5 to 9 drugs^c^ | 26,982 (34.4%) | 4,045 (34.0%) | 2,900 (32.7%) | 1,767 (33.5%) | 1,602 (32.5%) | 2,711 (32.7%) | 2,620 (32.6%) |
| 10 and more drugs^c^ | 25,062 (32.0%) | 3,745 (31.5%) | 2,831 (31.9%) | 1,339 (25.4%) | 1,284 (26.1%) | 2,352 (28.4%) | 3,562 (44.3%) |

^a^ Assessed any time prior to cohort entry.

^b^ Assessed in the 365 days before cohort entry.

^c^ Assessed in the 182 days before cohort entry.
